# Survey on *Mycoplasma hyopneumoniae* gilt acclimation practices in Europe

**DOI:** 10.1186/s40813-017-0069-y

**Published:** 2017-08-21

**Authors:** Laura Garza-Moreno, Joaquim Segalés, Maria Pieters, Anna Romagosa, Marina Sibila

**Affiliations:** 1 IRTA, Centre de Recerca en Sanitat Animal (CRESA, IRTA-UAB), Campus de la Universitat Autònoma de Barcelona, 08193 Bellaterra, Spain; 2 UAB, Centre de Recerca en Sanitat Animal (CRESA, IRTA-UAB), Campus de la Universitat Autònoma de Barcelona, 08193 Bellaterra, Spain; 3 Departament de Sanitat i Anatomia Animals, Facultat de Veterinària, UAB, 08193 Bellaterra, Spain; 4 Departament of Veterinary Population Medicine, College of Veterinary Medicine, University of Minnesota, St. Paul, MN 55108 USA; 5 PIC Europe, C/Pau Vila 22, 2° 6ª, 08174 Sant Cugat del Vallés, Barcelona, Spain

**Keywords:** *Mycoplasma hyopneumoniae*, Gilt acclimation, Survey, Questionnaire, Europe

## Abstract

Gilts are considered to play a key role in *Mycoplasma hyopneumoniae (M. hyopneumoniae)* transmission and control. An effective gilt acclimation program should ideally reduce *M. hyopneumoniae* shedding at first farrowing, decreasing pre-weaning colonization prevalence and potential respiratory problems in fatteners. However, information on gilt acclimation practices is scarce in Europe**.** The aim of this study was to identify current acclimation strategies for *M. hyopneumoniae* in Europe using a questionnaire designed to assess 15 questions focused on gilt replacement status, acclimation strategies and methods used to ascertain its effect. A total of 321 questionnaires (representing 321 farms) were voluntarily completed by 108 veterinarians (from 18 European countries). From these farms, 280 out of 321 (87.2%) were aware of the health status of gilts on arrival. From these 280 farms, 161 (57.5%) introduced *M. hyopneumoniae* positive replacements. In addition, 249 out of 321 (77.6%) farms applied an acclimation process using different strategies, being *M. hyopneumoniae* vaccination (145 out of 249, 58.2%) and the combination of vaccine and exposure to sows selected for slaughter (53 out of 249, 21.3%) the most commonly used. Notwithstanding, only 53 out of 224 (23.6%) farms, knowing the *M. hyopneumoniae* initial status and performing acclimation strategies against it, verified the effect of the acclimation by ELISA (22 out of 53, 41.5%), PCR (4 out of 53, 7.5%) or both (27 out of 53, 50.9%). This study showed that three fourths of the farms represented in this European survey have *M. hyopneumoniae* acclimation strategies for gilts, and one fifth of them verify to some extent the effect of the process. Taking into account that the assessment of acclimation efficacy could help in optimizing replacement gilt introduction into the breeding herd, it seems these practices for *M. hyopneumoniae* are still poorly developed in Europe.

## Background

*Mycoplasma hyopneumoniae (M. hyopneumoniae)* is the etiological agent of swine enzootic pneumonia (EP), a respiratory disease that mainly affects growing and finishing pigs [1]. Despite all efforts carried out to control this disease, such as vaccination and antimicrobial treatments, the economic losses caused by this pathogen are still a major concern to the swine industry [2].

*M. hyopneumoniae* is mainly transmitted by direct contact (nose-to nose) between pigs either horizontally (from infected to susceptible/naïve pigs) or vertically from sows to their piglets, although another putative indirect transmission route as aerosol has also been described [1–6]. Considering that intra-uterine transmission does not occur and that the maternally derived antibodies are not fully protective against infection, newborn piglets are susceptible to *M. hyopneumoniae* colonization [7]. Thus, contact between infected sows and their piglets may be the starting point of a subsequent chain of penmates transmission and, in consequence, a triggering factor for lung lesion development later on [8, 9]. Although old parity sows may also act as *M. hyopneumoniae* shedders, gilts are thought to be the main source of dam-piglet transmission [10, 11]. This, together with a low adjusted reproduction ratio (R_n_ = 1.16) [12] but persistent infection (up to 200 days post infection, dpi) [13], lead to the assumption that first farrowing might be a key point for controlling *M. hyopneumoniae* shedding [14]. Indeed, the absence of gilt acclimation (based on the contact of the replacement stock with other living animals) was classified, among other parameters, as an important risk factor for the disease severity [5, 7, 15]. Therefore, an adequate gilt acclimation focused on reducing the *M. hyopneumoniae* shedding by the sow at first farrowing should potentially decrease *M. hyopneumoniae* prevalence in piglets at weaning and the subsequent respiratory problems in fatteners [9, 14, 16].

Nowadays, information about *M. hyopneumoniae* gilt acclimation programs of the swine industry is very limited [17]; up to now there are two non-peer reviewed studies, one describing the situation in US [17] and another one in Mexico [18]. To the authors’ knowledge, there is no general data available on *M. hyopneumoniae* gilt acclimation protocols conducted in Europe. Therefore, this study was designed to expand the knowledge about replacement stock status in regards of *M. hyopneumoniae* and to identify the gilt acclimation strategies performed in the European continent.

## Methods

The survey on *M. hyopneumoniae* European gilt acclimation practices was based on a questionnaire. Such questionnaire was submitted by e-mail and/or by e-mail invitation with the web link (*https://es.surveymonkey.com/r/3QxMQ8Z*) to swine veterinarians across Europe. E-mail contacts were obtained from databases from *Centre de Recerca en Sanitat Animal* (CReSA), European College of Porcine Health Management (ECPHM) and European Association of Porcine Health Management (EAPHM). Collected data was the product of the voluntary participation of veterinarians and is reported in a descriptive fashion. Each questionnaire represented data from one single farm and was counted as such.

The questionnaire included 15 questions, 10 of them closed (e.g. yes/no or multiple choice questions) and 5 semi-closed (e.g. days of exposure to the acclimation strategy). In the first part of the questionnaire, information related with production system and herd size was requested. In the second part of the document, questions were focused to four main topics:

- *Farm status in regards M. hyopneumoniae infection*. The objective of this question was to ascertain the knowledge of practitioners about the status of their farms and how this status was assessed (by clinical signs, lung lesions, polymerase chain reaction [PCR], enzyme-linked immunosorbent assay [ELISA] and/or others).
- *Gilt replacement origin and status*. In this section, information concerning to type of replacement (own, purchased or mixed), age of replacement on arrival (days), frequency of replacement entrance into the farm (per year) and number of replacement animals (per entrance) was asked. Additionally, *M. hyopneumoniae* health status of gilts on arrival and the method used to check this status was requested.
- *Acclimation strategies and timing.* This section asked for the availability of isolation sites for gilt acclimation in the farms (yes or no), management practices used in these facilities (all-in-all-out [AIAO] sites or continuous flow [CF]), protocol applied (vaccines, live animals or others) and the time of exposure of animals to these strategies (if used).
- *Methods used to assess the effect of such strategies.* The potential verification of *M. hyopneumoniae* acclimation in the farms, as well as the assessment method (PCR or ELISA), was also demanded.

## Results

**Fig. 1:**
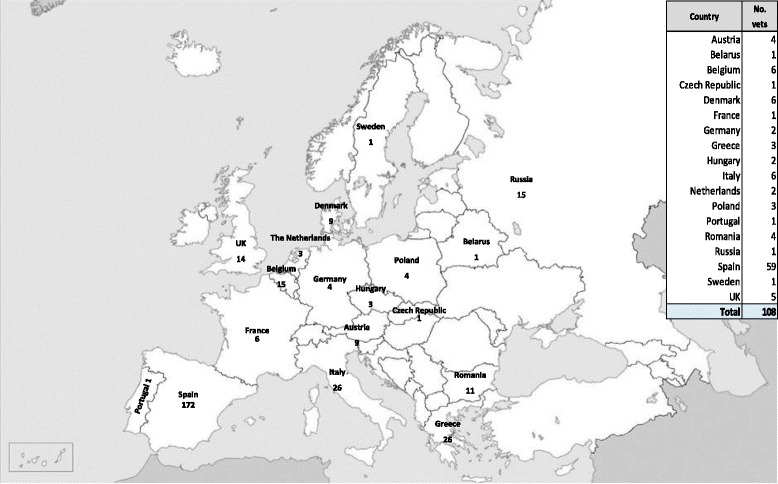
Number of questionnaires collected (*n* = 321) and number of responding veterinarians﻿ per European country

A total of 321 questionnaires were voluntarily completed by 108 veterinarians from 18 countries from the European continent (Fig. 1), representing globally 482,391 sows and 140,839 gilts. The median (Min-Max) number of questionnaires per veterinarian was 3.3 (1–15). General data of farms represented in the survey is shown in Table 1. From these 321 farms, 225 were from Southern European countries (Portugal, Italy, Spain and Greece; 70.1%) and 96 were from the rest of participant countries (29.9%).

**Table 1:** Number of farms included in the survey based on production system type and sow-farm size

Type of production system		n (%)	Size of sow farm		Number of gilts per sow farm	
			Median values	Range (Min-Max)	Median values	Range (Min-Max)
**One site**	Farrow to Finish	135 (42.1)	525	(75–7000)	130	(7–2450)
	Farrow to Wean	109 (34.0)	1000	(160–12,000)	352	(10–4400)
	Wean to Finish^a^	19 (5.9)	1200	(390–3500)	285	(50–1925)
	Finish^a﻿^	3 (0.9)	NR	NR	NR	NR
**Multiple sites**	Farrow to Wean + Wean to Finish	4 (1.2)	2000	(600–8000)	700	(100–4000)
	Farrow + Wean + Finish	51 (15.9)	1040	400–6500	400	(110–3380)
**Total**		321	NA	NA	NA	NA

*NA* Non-applicable, *NR* Non-reported

^a^Number of sows and gilts in this production system indicates the number of sows and gilts from those breeding farms where piglets came from

### Assessment of *M. hyopneumoniae* herd status

The assessment of *M. hyopneumoniae* farm status was reported by all but one of the farms (320 out of 321, 99.7%). Among these 320 farms, *M. hyopneumoniae* farm status was evaluated using one (80 [25.0%]), two (148 [46.3%]), three (51 [15.9%]), four (39 [12.2%]) and even five (2 [0.6%]) methods, respectively (Table 2).

**Table 2:** Number of farms (%) according to number of methods used by responders to assess *M. hyopneumoniae* status of farms

No. of methods	Methods					Total	Percent
	Clinical signs	Lung lesions	PCR	ELISA	Others		
0						1	0.3
1	✓					39	12.1
		✓				14	4.4
				✓		25	7.8
					✓	2	0.6
2	✓	✓				95	29.6
	✓			✓		22	6.9
	✓		✓			25	7.8
		✓		✓		4	1.2
	✓				✓	1	0.3
		✓	✓			1	0.3
3	✓		✓	✓		17	5.3
	✓	✓		✓		15	4.7
	✓	✓	✓			8	2.5
		✓	✓	✓		7	2.2
	✓	✓			✓	2	0.6
	✓			✓	✓	2	0.6
4	✓	✓	✓	✓		36	11.2
	✓	✓		✓	✓	2	0.6
		✓	✓	✓	✓	1	0.3
5	✓	✓	✓	✓	✓	2	0.6
Total	266	187	97	118	12	321	100.0

### Replacement origin and status

Approximately half of the surveyed farms introduced external replacement gilts (Table 3), whereas one-third used own replacement. The rest of the farms had a mixed (purchased + own) replacement practice. *M. hyopneumoniae* health status of replacement on arrival was known by 280 out of 321 (87.2%) farms, from which 161 (57.5%) were seropositive. Importantly, only 79 out these 280 (28.2%) farms confirmed the theoretical gilt status upon arrival. The most frequently used method to verify this status was ELISA (69 out of 79, 87.3%). Results of such verification were not requested.

**Table 3:** Number of farms (%) based on replacement origin, *M. hyopneumoniae* health status and the verification of replacement status

No. of farms according to the replacement source (%)	No. of farms according to *M. hyopneumoniae* health status (%)			No. of farms which verify the health status of the replacement (%)	Method used for replacement verification (%)			
					ELISA	PCR	ELISA + PCR	Others
Purchased 145 (45.2)	Known 126 (86.9)	Positive	61/126 (48.4)	4/61 (6.6)	2/4 (50.0)	0/4 (0.0)	2/4 (50.0)	0/4 (0.0)
		Negative	36/126 (28.6)	10/36 (27.8)	8/10 (80.0)	1/10 (10.0)	1/10 (10.0)	0/10 (0.0)
		Non-specified	29/126 (23.0)	24/29 (82.8)	24/24 (100.0)	0/24 (0.0)	0/24 (0.0)	0/24 (0.0)
	Unknown 19 (13.1)	NA	NA	NA	NA	NA	NA	NA
Own 103 (32.1)	Known 86 (83.5)	Positive	60/86 (69.8)	12/60 (20.0)	10/12 (83.3)	2/12 (16.7)	0/12 (0.0)	0/12 (0.0)
		Negative	25/86 (29.1)	22/25 (88.0)	21/22 (95.5)	0/22 (0.0)	0/22 (0.0)	1/22 (4.5)
		Non-specified	1/86 (1.1)	0/86 (0.0)	NA	NA	NA	NA
	Unknown 17 (16.5)	NA	NA	NA	NA	NA	NA	NA
Mixed 73 (22.7)	Known 68 (93.2)	Positive	40/68 (58.8)	2/40 (5.0)	1/2 (50.0)	0/2 (0.0)	0/2 (0.0)	1/2 (50.0)
		Negative	27/68 (39.7)	4/27 (14.8)	3/4 (75.0)	1/4 (25.0)	0/4 (0.0)	0/4 (0.0)
		Non-specified	1/68 (1.5)	1/1 (100.0)	0/1 (0.0)	0/1 (0.0)	0/1 (0.0)	1/1 (100.0)
	Unknown 5 (6.8)	NA	NA	NA	NA	NA	NA	NA

*NA* non-applicable

**Fig. 2:**
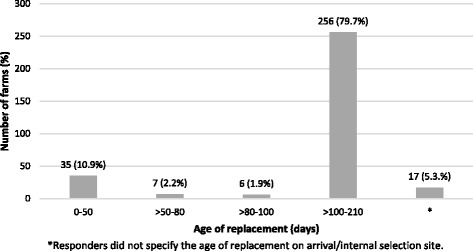
Number of farms (%) included in the survey according to the age of replacement gilts on arrival/internal selection site (days)

**Fig. 3:**
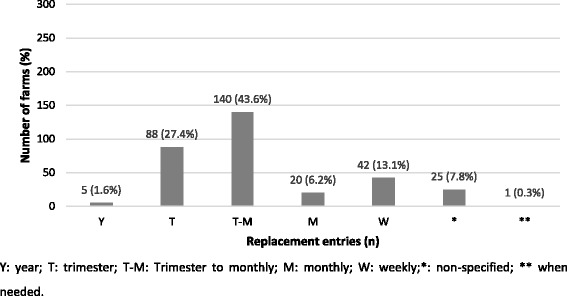
Number of farms (%) based on the frequency of replacement entry into farms included in the survey

Age of replacement on arrival at sow farm (in case of farms with purchased replacement) or at internal selection site (in case of farms with own replacement) differed among studied farms, varying from 0 to 210 days (Fig. 2), being animals older than 100 days the most frequent one. Frequency of replacement batch entry into the herd also varied, from annual to weekly or as needed without further specification (Fig. 3). Gilt introduction every three or four months was the most frequent practice (228 out of 321, 71.0%).

### Acclimation strategies

Information of *M. hyopneumoniae* acclimation strategies performed is shown in Table 4. From the 321 farms for which information was obtained in the questionnaire, 278 (86.6%) had gilt isolation sites available for acclimation, which were managed mainly by AIAO practices. From these 278 farms, 225 (80.9%) farms, performed a specific acclimation process for *M. hyopneumoniae.* From these 225 farms, 159 (70.7%) were located in Southern European countries and 66 (29.3%) in the rest of European participating countries. However, there were 24 farms that, although not having isolation units or not answering this question, performed acclimation process for *M. hyopneumoniae* (no more information was available regarding the specific site used to acclimate gilts). Additionally, from these 249 farms performing acclimation strategies, *M. hyopneumoniae* status of gilts on arrival was investigated in 224 farms (224/249, 90.0%).

**Table 4:** Information of *M. hyopneumoniae* acclimation strategies performed by the respondents

	Availability of isolation units (%)	Management practices in isolation units				No. of farms performing *M. hyopneumoniae* acclimation strategies (%)	Mean (range) of duration of acclimation period (days)	No. of farms verifying acclimation (%)	Methods used for acclimation verification (%)		
		AIAO	CF	AIAO/CF	NR				ELISA	PCR	ELISA + PCR
Collected data	Yes 278 (86.6)	122/278 (43.9)	82/278 (29.5)	1/278 (0.4)	73/278 (26.2)	224/278 (80.6)	28.3 (7–180)	53/224 (23.7)	22/53 (41.5)	4/53 (7.5)	27/53 (50.9)
	No 32 (10.0)	NA	NA	NA	NA	19/32 (59.4)	37 (21–60)	0/19 (0.0)	NA	NA	NA
	Unknown 11 (3.4)	NA	NA	NA	NA	5/11 (45.5)	NR	0/5 (0.0)	NA	NA	NA
Total	321	122	82	1	73	249	NA	54	22	4	27

*AIAO* all in-all out, *CF* continuous flow, *NR* non-reported, *NA* non-applicable

Vaccination against *M. hyopneumoniae* was the most used acclimation procedure (145/249, 58.2%), followed by vaccination and animal exposure, with either culled sows (53/249, 21.3%) or pigs (13/249, 5.2%), and a combination of vaccination and both types of live animal exposure (31/249, 12.4%) (Table 5). The number of vaccine doses used during gilt acclimation varied among one (71 out of 243, 29.2%), two (77 out of 243, 31.7%) or three (38 out of 243, 15.6%). There were 57 (23.4%) farms for which no data were available on how many doses were administered to gilts.

**Table 5:** Number of farms (%) according the methods used for replacement gilt acclimation in terms of *M. hyopneumoniae*

No. methods	Vaccination	﻿Exposure to﻿ selected sows for slaughter	Exposure to pigs	Others	Total	%
0					72	22.4
1	✓				145	45.2
		✓			2	0.6
			✓		1	0.3
				✓	1	0.3
2	✓	✓			53	16.5
	✓		✓		13	4.1
		✓	✓		2	0.6
	✓			✓	1	0.3
3	✓	✓	✓		31	9.7
Total	243	88	47	2	321	100.0

*M. hyopneumoniae* specific acclimation was performed by 76.4% (172/225) and 80.2% (77/96) of farms from Southern and rest of Europe, respectively. In both geographic regions vaccination was the most frequently used method, reaching values of around of 95%. Nevertheless, whereas in Southern regions combined vaccination with animal exposure (cull sows and/or pigs) was practiced, farms located in the rest of Europe mainly used vaccination alone.

Finally, considering the farms where the initial status was known and the acclimation process was carried out, the verification of the effect of such strategies was performed in only 23.7% of the farms (53/224), being the combination of ELISA and PCR the most frequently used method. Importantly, the confirmation of theoretical gilt status upon arrival and after gilt acclimation to verify the effect was checked in 49.4% farms (39/79). Results of such verification were not requested.

## Discussion

The aim of the present study was to gain knowledge on *M. hyopneumoniae* gilt status and acclimation practices conducted in European farms by means of a descriptive study. To date, no information about this issue is available in Europe and, in fact, minimal data do exist from around the world, with surveys performed in USA [17] and Mexico [18].

Questionnaires were voluntarily responded, thus, they may have some inherent biases in the responses [19]. Indeed, in this investigation, most of the veterinarians (70.7%) were from Southern European countries. Representation of Central and Northern European countries was more limited; this was already expected for some Scandinavian countries due to the low or no prevalence of *M. hyopneumoniae* in pig farms (O. Peltoniemi, Finland, and Carl-Andreas Grøntvedt, Norway, personal communications). Although the European situation might not be fully represented (especially regarding small pig farms with non-specialized swine veterinarians), no significant differences in terms of gilt acclimation practices were detected between the South of Europe (Portugal, Spain, Italy and Greece) and the rest of participating countries (data not shown). Nevertheless, information obtained about *M. hyopneumoniae* current status on gilt acclimation should help depicting measures that can be potentially applied elsewhere. Additionally, the facts that all but one of the participants evaluated *M. hyopneumoniae* status of their farm and more than 80% were aware of their replacement status regarding this pathogen, suggest that EP is still a concern to swine industry. Notwithstanding, this assumption could be influenced by the fact that only concerned veterinarians on *M. hyopneumoniae* completed the questionnaire.

Most of the questionnaire respondents reported that the assessment of *M.hyopneumoniae* associated problems was based on presence of clinical signs accompanied with lung lesion scoring at slaughterhouse. Noteworthy, non-productive dry coughing and cranio-ventral pulmonary consolidation (CVPC), the usual clinical signs and lung lesions attributed to *M. hyopneumoniae* infection, can also be produced by other respiratory pathogens [2, 20], and these parameters do not allow detecting a potential subclinical infection. In consequence, clinical disease assessment should be supplemented with the laboratory confirmation of *M. hyopneumoniae* involvement in clinical signs and lesions [16]. A total of 151 out of 320 (47.1%) farms in which *M. hyopneumoniae* status was evaluated based their assessment only on non-specific methods (clinical signs or lung lesions scoring at abattoir), suggesting that most European farms represented in this study performed an incomplete assessment of *M. hyopneumoniae* health status. This percentage is lower than Mexican survey (no data is available for US), since in that study, 71% of the respondents evaluated *M. hyopneumoniae* farms situation only according to clinical signs [18].

The introduction of external replacement into a swine herd is considered a potential risk of new pathogen introduction and farm health destabilization [21, 22], as well as for becoming infected or re-infected with different strains [5, 15]. However, more than 40% of the evaluated farms purchased external replacement, being in most of the cases seropositive against *M. hyopneumoniae*. Comparatively, percentage of positive replacement in the assessed European farms was similar (161/280, 57.5%) to that in the USA (55%), but lower than in Mexico (90%) [17, 18].

Interestingly, most farms (80.9%) had isolation facilities to acclimate, being the most utilized the AIAO system. In terms of type of management systems used to acclimatize gilts, a clear difference between participating European farms, USA and Mexico was observed. Whereas 75% and 72% of Mexican [18] and USA [17] farms used continuous flow to acclimatize gilts, respectively, only 29.5% of European farms of the present survey performed such strategy. These differences probably reflect the different production systems used in each country.

Replacement gilt acclimation methods used in Southern European farms were mainly based on vaccination alone or in combination with live animal exposure (culled sows or pigs), whereas farms from rest of participant countries utilized vaccination exclusively. These results were in line with the ones reported by USA and Mexican studies [17, 18], in the sense that vaccination was the most used approach. The rationale behind this strategy would be linked to the reduction of the number of animals showing CVPC, reduction of clinical signs (coughing) and decrease of number of microorganisms and bacterial shedding [23]. However, current vaccines against *M. hyopneumoniae* are not able to prevent bacterial colonization and the transmission between vaccinated pigs seems not to be significantly altered [21, 24–27]. Presumably infected culled sows or pigs were utilized as potential *M. hyopneumoniae* shedders in 27.4% and 14.6% of studied farms, respectively; however their shedding status was not required in the questionnaire. These results are in agreement with previous studies in USA (34% of respondents utilized culled sows to acclimatize) and Mexico (27% of responders used culled sows and 10% piglets) [17, 18]. Acclimation strategies based on others (unspecified methods) were very scarce (0.3%).

Finally, the relatively low percentage of farms verifying the acclimation process (23.7%) indicated that most of the surveyed farms did not evaluate gilt infection and shedding status at first farrowing. This situation coincided with the information reported by Mexican and USA studies, where only 20% and 14% of responders, respectively, validated the acclimation process. An inadequate acclimation process could imply that gilts would be a potential source of infection for its offspring and, therefore, leading to an outbreak of *M. hyopneumoniae* in seronegative farms or *M. hyopneumoniae* re-circulation/re-infection in seropositive ones.

## Conclusions

The present study shows that most of the European farms introduced *M. hyopneumoniae* positive replacement stock, but only a minority assessed its health status on arrival. Likewise, most of participating farms performed a specific gilt acclimation procedure against *M. hyopneumoniae*. Moreover, the verification of this process was not a common practice.

## Abbreviations

CVPC: cranio-ventral pulmonary consolidation
ELISA: Enzyme-linked immunosorbent assay
*M. hyopneumoniae*: *Mycoplasma hyopneumoniae*
PCR: Polymerase chain reaction
